# Polyandry enhances offspring viability with survival costs to mothers only when mating exclusively with virgin males in *Drosophila melanogaster*

**DOI:** 10.1002/ece3.3152

**Published:** 2017-08-14

**Authors:** Sergio Castrezana, Brant C. Faircloth, William C. Bridges, Patricia Adair Gowaty

**Affiliations:** ^1^ Department of Ecology and Evolutionary Biology University of California, Los Angeles Los Angeles CA USA; ^2^ Institute of the Environment and Sustainability University of California, Los Angeles Los Angeles CA USA; ^3^ Smithsonian Tropical Research Institute Washington DC USA; ^4^ Department of Mathematical Sciences Clemson University Clemson SC USA

**Keywords:** *Drosophila melanogaster*, female lifespan, monogamy, multiple mating, offspring viability

## Abstract

A prominent hypothesis for polyandry says that male–male competitive drivers induce males to coerce already‐mated females to copulate, suggesting that females are more likely to be harassed in the presence of multiple males. This early sociobiological idea of male competitive drive seemed to explain why sperm‐storing females mate multiply. Here, we describe an experiment eliminating all opportunities for male–male behavioral competition, while varying females’ opportunities to mate or not with the same male many times, or with many other males only one time each. We limited each female subject's exposure to no more than one male per day over her entire lifespan starting at the age at which copulations usually commence. We tested a priori predictions about relative lifespan and daily components of RS of female *Drosophila melanogaster* in experimental social situations producing lifelong virgins, once‐mated females, lifelong monogamous, and lifelong polyandrous females, using a matched‐treatments design. Results included that (1) a single copulation enhanced female survival compared to survival of lifelong virgins, (2) multiple copulations enhanced the number of offspring for both monogamous and polyandrous females, (3) compared to females in lifelong monogamy, polyandrous females paired daily with a novel, age‐matched experienced male produced offspring of enhanced viability, and (4) female survival was unchallenged when monogamous and polyandrous females could re‐mate with age‐ and experienced‐matched males. (5) Polyandrous females daily paired with novel virgin males had significantly reduced lifespans compared to polyandrous females with novel, age‐matched, and experienced males. (6) Polyandrous mating enhanced offspring viability and thereby weakened support for the random mating hypothesis for female multiple mating. Analyzes of nonequivalence of variances revealed opportunities for within‐sex selection among females. Results support the idea that females able to avoid constraints on their behavior from simultaneous exposure to multiple males can affect both RS and survival of females and offspring.

## 1 INTRODUCTION

Classical ideas (Bateman, 1948) say females are “passive” and choosy with limited reproductive capacities predicting few benefits for polyandrous females. Yet, polyandry is common (Anderson, 1974; Gowaty, 2006, 2012, 2013; Gowaty & Hubbell, 2013; Taylor, Price, & Wedell, 2014), despite potential fitness costs to females (Otti, 2015). Given classical assumptions, several functional hypotheses may explain why females multiply mate including that female multiple mating may be a result of male–male competitive drive (Trivers, 1972), occurring when males coerce females to mate. Male manipulation (“gifts” or “lures”) may affect females’ nutritional status enhancing direct benefits for females whenever males provide females with resources transferred during courtship or mating (Arnqvist & Nilsson, 2000), but male coercive mechanisms by definition also create female fitness costs. Female multiple mating may also arise as a correlate to selection on males to mate multiply (Halliday & Arnold, 1987), and it is possible that endosymbionts manipulate females to mate multiply (Wedell, 2013).

**Table 1:** Experimental treatments

Treatment	Social manipulation and sample size
*V*_L_	Female virgins alone for life (*n* = 30).
*V*_LM_	Male virgins alone for life (*n* = 30).
*M*_OC_	Females (*n* = 30) with one male for one day then alone until death; after one day with a female males (*n* = 30) were held for life in separate vials.
*M*_L_	Females (*n* = 30) were with the same, same‐aged male (*n* = 30) for life, and copulated ad libitum.
*P*_V_	Females (*n* = 30) with a novel male each day: a new, young, inexperienced virgin male, with copulation ad libitum. We discarded virgin males after one day with the subject females.
*P*_E_	Females (*n* = 30) with a novel, experienced male (*n* = 30), each age‐matched‐to‐females with copulation ad libitum. We round‐robin rotated males daily. For example, on day 2 female 1 was with the male who was with female 2 on day 1. On day 3, female 1 was with the male who was with female 2 on day 2, and so‐forth. Because of death day variation, we held some females or males for a single day without exposure to the opposite sex.

An alternative (Anderson, Kim, & Gowaty, 2007; Gowaty, 1996, 2008; Gowaty et al., 2007b) to classical ideas that females are coy and choosy assumes that females have evolved resistance mechanisms to coercion, whenever coercion is costly to female fitness or the fitness of their offspring. For example, whenever wild‐living females are able to escape or avoid the behavioral effects of male harassment (Gowaty, 1996), polyandry may evolve with few costs to breeding females and with health or other viability benefits for offspring (Gowaty, 2008; Gowaty, Kim, Rawlings, & Anderson, 2010; Lively, 1996; Simmons & Holley, 2011). The freedom of females’ movements in wild flies is notable to watchers in the wild (Markow & O'Grady, 2005), particularly so for females that first arrive at new feeding sites (SC *pers obs*). In the wild, flying females may be able to escape or avoid coercive males, just because they can fly away. However, in the general discussion of potential mechanisms affecting female reproductive decisions–including to mate or not with multiple males under coercion–investigators seldom focus on females’ options to avoid coercion, which is what we have attempted to do here, while testing a variety of potential explanations for female multiple mating.

Given the diversity of the hypotheses explaining multiple mating and recently reviewed in Gowaty (2012; 2013), we used an experimental design (Table 1) allowing simultaneous tests of alternative predictions of multiple hypotheses, while reducing opportunities for male behavioral coercion of females. Using captive *Drosophila melanogaster* free of *Wolbachia* and *Spiroplasma* endosymbionts, we controlled females’ exposure to conspecific males so that no female in any treatment was with more than one male in a single day, providing some leveling of the ecological playing field of subjects in a way that seldom occurs in captive studies (Billeter, Jagadeesh, Stepek, Azanchi, & Levine, 2012; Maklakov, Immler, Løvlie, Flis, & Friberg, 2013).

We report variation in components of fitness of female subjects in two by two matched sets of treatments testing a priori predictions (Leek & Peng, 2015) of hypotheses (Gowaty, 2012, 2013; Gowaty et al., 2010) about the fitness costs/benefits of mating opportunities available to female subjects. Predictions include the following:

1. Females may gain direct fitness benefits from exposure to males in which case virgins may die faster than mated females.
2. Limits to the number of, or the viability of sperm in a single ejaculate, probably occasionally occur in most organisms, especially those without sperm‐storing tissues or organs, but flies do have “sperm management” organs (Markow & O'Grady, 2005) suggesting that one copulation for many organisms is enough to fertilize a females’ lifelong production of eggs. Nonetheless, sperm limitation occurs in some Drosophila species (Turner & Anderson, 1983, 1984) and may favor female multiple mating in *D. melanogaster*. If so, females achieving only a single copulation may oviposit fewer eggs than females with multiple copulations.
3. If multiple mating increases female exposure to pathogens or parasites (Lively, 1996), female lifespan may be reduced.
4. However, even if polyandry extracts costs decreasing female survival, mating with multiple males may allow them to increase reproductive success (RS) through sorting of male haplotypes in a lottery competition (Williams, 1975) enhancing the health of offspring thereby increasing lineage success (i.e*.,* grand‐descendants) (Price, Hurst, & Wedell, 2010).
5. If 4) is so, polyandrous females may have shorter lifespans, but higher reproductive success than lifelong monogamous females.
6. Female multiple mating may occur because of male–male competitive drive resulting in behavioral coercive polyandry. If so, when females can escape or otherwise mitigate coercive polyandry, females may manage their re‐mating schedules to reap fitness rewards that may accrue without incurring costs.
7. Under the assumption that experienced males, age‐matched to females are less “eager” and less coercive than young, virgin males (Hoffmann, 1990) (which has been attributed to conditioning after male exposure to already‐mated females), polyandrous females with age‐matched males may live longer than polyandrous females exposed daily to young, virgin males, and perhaps as long as females in lifelong monogamy.
8. Polyandry may be a correlated response to selection on males to mate with multiple females. If so, female costs likely accumulate given exposure to pathogens reducing female lifespan, but without effects on female reproductive success.

If these predictions about RS and survival are met, the results would indicate consistency with the hypotheses. When the predictions are not met, the results would indicate inconsistency with the hypotheses. Keep in mind that consistency does not rule out consistency with other hypotheses. Inconsistency with the predictions, however, would be useful for inferences related to adaptive significance.

**Figure 1:**
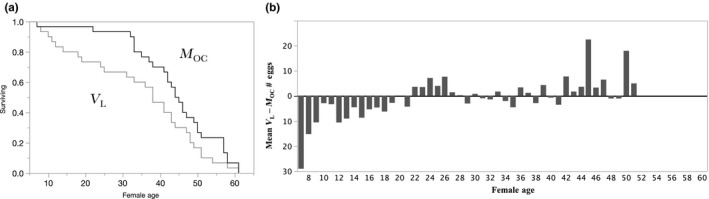
*M*_OC_ versus *V*_L_ females. (a) *M*_OC_ females lived significantly longer than *V*_L_ females (Log‐Rank = 3.1520, *df* = 1, *P* > Chi‐square = 0.0758; Wilcoxon 4.4467, *df* = 1, *P* > Chi‐square = 0.0350). (b) Daily mean difference scores of matched pairs number of eggs show that on average *M*_OC_ females laid 2.6 ± 0.977 (*SE*) more eggs/day than *V*_L_ females, and *M*_OC_ females laid more eggs than *V*_L_ females on most days of life (Wilcoxon Signed Rank *S* = −318, 49, *P* > |*S*| < 0.0015 and *P* < *S* = 0.0007)

**Figure 2:**
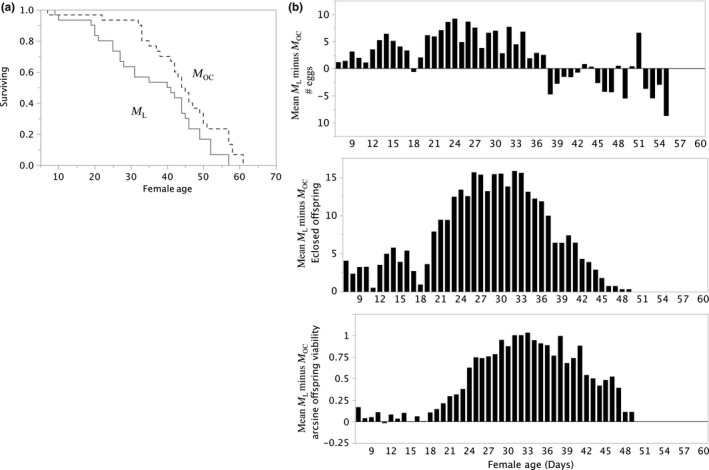
*M*_OC_ versus *M*_L_ female survival (a) and components of RS (b). (a) Product‐limit survival fit of *M*_OC_ versus *M*_L_ females shows significant differences (Log‐Rank *X* ^2^ = 4.6546, *df* 1, *P *> *X* ^2^ = 0.031; Wilcoxon *X* ^2^ = 4.7046, *df* 1, *P *> *X* ^2^ = 0.030. (b) *M*_L_–*M*_OC_ matched pairs means by female ages (*N* = 49) in components of RS: *Top panel: number of eggs*:* M*_L_ oviposited 2.07 ± 0.63 (*SE*) more eggs/day than *M*_OC_ females (Wilcoxon Signed Rank *S* = 302.5, *df* 48, *P* > |*S*| < 0.0018 and *P* > *S* = 0.0009). *Middle panel: number of eclosed offspring*. *M*_L_ females had 6.5 ± 0.78 (*SE*) more eclosed offspring than *M*_OC_ females (Wilcoxon Signed Rank *S* = 473, *P* > |*S*| < 0.0001 and *P *> *S* = 0.0001). *Bottom panel: arcsine fraction egg‐to‐adult survival*. *M*_L_ females’ average egg‐to‐adult survival was 0.419 ± 0.05 (*SE*) greater than *M*_OC_ females (Wilcoxon Signed Rank *S* = 469.000, *P* > |*S*| < 0.0001 and *P *> *S* = 0.0001)

**Figure 3:**
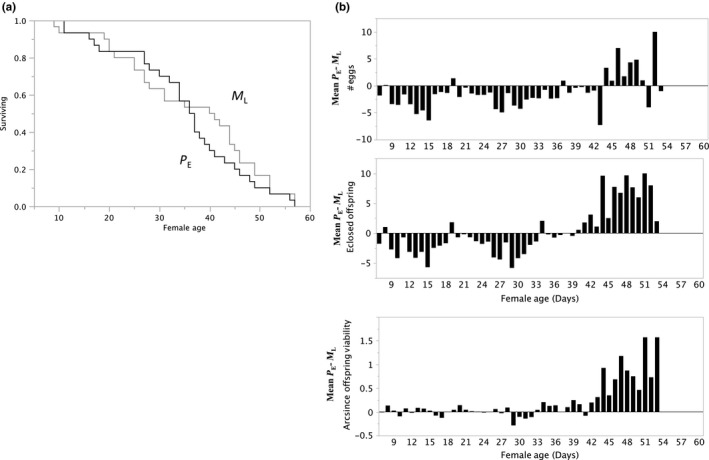
*M*_L_ versus *P*_E_ of female survival (a), and (b) components of RS for females. (a) Product‐limit survival fit of *M*_L_ versus *P*_E_ shows no statistically significant differences (Log‐Rank *X* ^2^ = 0.6576, *df* 1, *P *>* X* ^2^ = 0.4174; Wilcoxon *X* ^2^ = 0.2036, *df* 1, *P *> *X* ^2^ = 0.6518. (b) *P*_E_–*M*_L_ matched treatment sets mean differences by female ages (*N* = 47) in components of RS: *Top panel: number of eggs*:* M*_L_ oviposited 1.2 ± 0.47 (*SE*) more eggs/day than *P*_E_ females (Wilcoxon Signed Rank *S* = −296.50, *df* = 46, *P* > |*S*| < 0.0011 and *P* < *S* = 0.0005). *Middle panel: number of eclosed offspring*. *P*_E_ females had 15.7 eclosed offspring/day and *M*_L_ females had 15.4, with a mean difference of 0.33 ± 0.61 (*SE*) offspring (Wilcoxon Signed Rank *S* = −40.000, *P* > |*S*| < 0.6768). *Bottom panel: arcsine fraction egg‐to‐adult survival*. *P*_E_ females’ average egg‐to‐adult survival was ±0.22 ± .06 (*SE*) greater than *M*_L_ females (Wilcoxon Signed Rank *S* = 331.000, *P* > |*S*| = 0.0002 and *P* > *S* = 0.0001)

**Figure 4:**
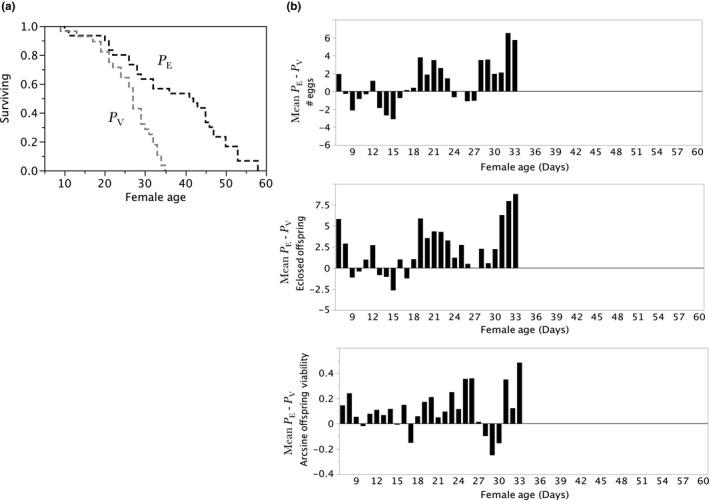
*P*_V_ versus *P*_E_ comparisons of female survival (a) and components of RS (b). (a) Product‐limit survival fit of *P*_V_ versus *P*_E_ shows statistically significant differences in female lifespan (Log‐Rank *X* ^2^ = 27.2171, *df* 1, *P *>* X* ^2^ = 0.0001; Wilcoxon *X* ^2^ = 18.6104, *df* 1, *P *>* X* ^2^ = 0.0001). (b) *P*_E_–*P*_V_ matched pairs mean differences over female age (*N* = 27) in components of RS. *Top panel: number of eggs*:* P*_E_ oviposited 0.94 ± 0.47 (*SE*) more eggs/day than *P*_V_ females (Wilcoxon Signed Rank *S* = 69.00, *P* < *S* = 0.0490). *Middle panel: number of eclosed offspring*. *P*_E_ females had 2.27 ± 0.56 (*SE*) more eclosed offspring/day than *P*_V_ females (Wilcoxon Signed Rank *S* = .000137, *P* > |*S*| < 0.0003; Prob > *S* = 0.0001). *Bottom panel: arcsine fraction egg‐to‐adult survival*. *P*_E_ females’ average/day arcsine egg‐to‐adult survival was 1.19766 and *P*_V_ females was 1.09 (Wilcoxon Signed Rank *S* = 117.000, *P* > |*S*| = 0.0030 and *P* < *S* = 0.0015)

**Figure 5:**
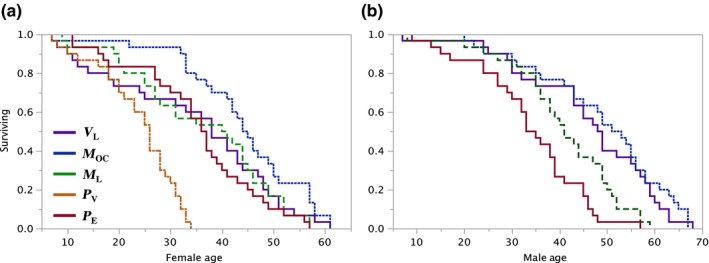
Lifespan variation by treatment differed significantly among females (a) and among males (b). (a) Log‐Rank *X* ^2^ = 37.97, *df* = 4, *p* < .0001; Wilcoxon = 44.1, *df* = 4, *p* < .0001; (b) Log‐Rank *X* ^2^ = 34.0586, *df* = 4, *p* < .0001; Wilcoxon = 24.9556, *df* = 3, *p* < .0001. Contrast analyzes for a priori planned tests between females are in Figures 1, 2, 3, 4

**Figure 6:**
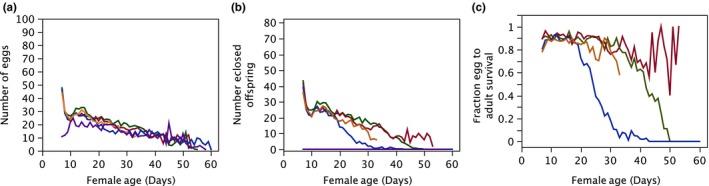
Means/day by treatment of components of reproductive success over female lifespan. *V*_F_, violet; *M*_OC_, blue; *M*_L_, green; *P*_E_, red; *P*_V_, orange

Table 1 shows treatments and sample sizes. Figures 1, 2, 3, 4 display comparisons between treatments for testing specific a priori hypotheses. Figure 1 shows results comparing lifelong virgins (*V*_LF_) to females exposed to one male on only 1 day (*M*_OC_ **).** Figure 2 shows results comparing *M*_OC_ females‐to‐females in lifelong monogamy (*M*_L_). Figure 3 shows results comparing *M*_L_ females‐to‐*P*_E_ females in lifelong polyandry who were exposed to a novel, age‐matched male each day. Figure 4 has results comparing *P*_E_ females to *P*_V_ females exposed to a young virgin male each day. Table 2 summarizes the predictions and results of a priori planned tests (Leek & Peng, 2015) of each hypothesis. We also performed unplanned exploratory analyzes (Leek & Peng, 2015): (a) over all treatments combined of lifespan variation of females (Figure 5a) and males (Figure 5b; (b) mean changes over female lifespan in components of female reproductive success (Figure 6); and (c) of between‐treatment variances in female RS and survival (Table 3) facilitating a discussion of the opportunity for selection on females in the absence of behavioral sexual selection in either males or females.

**Table 2:** *A priori* planned tests of predictions (second column) and results of tests (third column) of hypotheses of adaptive significance of multiple copulations and polyandry

Polyandry hypotheses Components of fitness	Predicted	Observed
Ejaculate contributions nourish zygotes and females or otherwise induce advantageous‐to‐females physiology		
Eggs oviposited	*V*_L_ < *M*_OC_	*V*_L_ < *M*_OC_
Eclosed adult offspring	Silent	
Egg‐to‐adult viability	Silent	
Mother longevity	*V*_L_ < *M*_OC_	*V*_L_ < *M*_OC_
Multiple copulations guard against inadequate or inviable sperm		
Eggs oviposited	*M*_OC_ < *M*_L_	*M*_OC_ < *M*_L_
Eclosed adult offspring	*M*_OC_ < *M*_L_	*M*_OC_ < *M*_L_
Egg‐to‐adult viability	*M*_OC_ < *M*_L_	*M*_OC_ < *M*_L_
Mother longevity	Silent	*M*_OC_ > *M*_L_
Polyandry enhances offspring viability		
Eggs oviposited	Silent	*M*_L_ > *P*_E_
Eclosed adult offspring	Silent	*M*_L_ = *P*_E_
Egg‐to‐adult viability	*M*_L_ < *P*_E_	*M*_L_ < *P*_E_
Mother longevity	Silent	*M*_L_ = *P*_E_
Correlated response to selection on males to mate multiply with the auxiliary hypothesis that multiple mates increase female's exposure to pathogens		
Eggs oviposited	*M*_L_ = *P*_E_	*M*_L_ > *P*_E_
Eclosed adult offspring	*M*_L_ = *P*_E_	*M*_L_ = *P*_E_
Egg‐to‐adult viability	*M*_L_ = *P*_E_	*M*_L_ < *P*_E_
Mother longevity	*M*_L_ > *P*_E_	*M*_L_ = *P*_E_
Male–male competitive drive produces polyandry with greater sexual conflict reducing female survival		
Eggs oviposited	*P*_E_ ≤ *P*_V_	*P*_E_ > *P*_V_
Eclosed adult offspring	*P*_E_ ≤ *P*_V_	*P*_E_ > *P*_V_
Egg‐to‐adult viability	*P*_E_ ≤ *P*_V_	*P*_E_ > *P*_V_
Mother longevity	*P*_E_ > *P*_V_	*P*_E_ > *P*_V_

**Table 3:** Exploratory tests of inequality of variances in components of fitness by treatments

Treatment	Female lifespan (days) ***^a^	# Eggs/day*^b^	# Eclosed offspring/day^c^	Development time (days)^d^	Fraction Egg‐to‐adult survival/female***^e^
*V*_L_	35.7 ± 16.1	10.7 ± 7.5	0		
*M*_OC_	45.7 ± 12.5	14.6 ± 3.8	13.1 ± 5.1	9.35 ± 0.34	0.56 ± 0.21
*M*_L_	38 ± 14.1	21.83 ± 6.5	21.9 ± 7.2	9.45 ± 0.29	0.85 ± 0.14
*P*_V_	25 ± 7.6	21.76 + 6.5	22.5 ± 10.8	9.25 ± 0.36	0.85 ± 0.16
*P*_E_	36.9 ± 12.6	19.9 ± 6	21 ± 5.8	9.42 ± 0.38	0.88 ± 0.06

a Brown‐Forsythe *F*‐ratio = 3.6858, *df* = 4, *P* > *F* = 0.0068; Levene *F*‐ratio = 4.8575, *df* = 4, *P* > *F* = 0.0010.

b Brown‐Forsythe *F*‐ratio = 2.66, *df* = 4, *P* > *F* 0.0348; Levene *F*‐ratio = 3.1854, *df* = 4, *P* > *F* = 0.0153.

c Brown‐Forsythe, *F*‐ratio = 1.269, *df* 3, *P* > *F* = 0.2883; Levene, *F*‐ratio = 1.474, *df* = 3, *P* > *F* = 0.2252.

d Brown‐Forsythe, *F*‐ratio = 0.6103, *df* 3, *P* > *F* = 0.6093; Levene, *F*‐ratio = 0.8544, *df* = 3, *P* > *F* = 0.4664.

e Brown‐Forsythe, *F*‐ratio = 7.4, *df* 3, *P* > *F* = 0.0001; Levene, *F*‐ratio = 8,29, *df* = 3, *P* > *F* = 0.0001.

## 2 METHODS

### 2.1 Notes on natural history

*Drosophila melanogaster* are common, human commensals. Females sometimes fail to re‐mate for about 5 days (Markow & O'Grady, 2005), but some females also may copulate several times in a single day before becoming refractory (SC *pers obs*).

### 2.2 Capture of flies and testing for endosymbionts

Our subjects were from a multifemale stock LA1206 set up in December, 2011 that included only individuals drawn from endosymbiont‐free isofemale lines that we collected between September and October 2010 from locations in Los Angeles, CA (Castrezana, Faircloth, & Gowaty, 2010). Before constituting LA1206, we tested 277 isolines (Braig, Zhou, Dobson, & O'Neill, 1998; O'Neill, Giordano, Colbert, Karr, & Robertson, 1992; Pool, Wong, & Aquadro, 2006) from over 200 locations in the Los Angeles basin. Fewer than 1% (nine isolines) were free of *Spiroplasma* and *Wolbachia*, although these lines where each collected at geographically distinct places in the Los Angeles area.

### 2.3 Culturing of subjects

We maintained the endosymbiont‐free isolines on cornmeal transferring them every 10–15 days for 19 months. In June 2012, we populated a “bug dorm” (Bioquip catalog #1462W) with 20 female and 20 male virgins from each of the endosymbiont‐free isolines and allowed this source population of 360 flies to expand for 3 months, or about 9–15 or more generations thus allowing for considerable genetic mixing of the isolines. Each week we replaced the bug‐dorm's 8 oz bottles containing 100 ml of cornmeal. Adult flies move freely in “bug dorms” (“bug dorm tents” have a volume slightly less than a cubic meter which can hold a huge number of flies). Adult females ovipoisited in bottles containing cornmeal placed in the bug dorm. On 9/1/2012, we removed all adult flies (>10,000 individuals) from the mass population of the bug dorm. On 9/2/2012 between 6 and 7 a.m., we collected, using a mouth aspirator, 150 newly eclosed virgins of each sex from bottles in the bug dorm, and placed each alone in a vial with 2 ml of cornmeal. We never used CO_2_ when handling flies. We expected that our culturing methods, including the expansion of the population, had allowed for a near‐natural level of genetic diversity among our subjects with limited opportunities for selection at least compared to other studies of polyandry in other captive insects.

### 2.4 Environmental conditions of the experimental room

We ran our experiments in a controlled‐temperature (21°C) room with 12/12‐hr light/dark period**.** UCLA's Drosophila Kitchen provided cornmeal fly food, which we modified: Using a BPA‐free container, we put 450 g of solidified cornmeal food and 100 ml ddH_2_O, which we melted in a microwave (high for 4.5 min), and then added 10 ml of alcohol (Everclear, 190‐proof, 95% ABV) as an extra mold inhibitor (SC *pers. obser*.). We used a 100 cc syringe to set 7.5 ml of cornmeal food in each vial.

### 2.5 Experimental controls

- Technicians and laboratory helpers were blind to our hypotheses and predictions.
- Once adult subjects were in vials, they were never able to encounter another same sex individual. Thus, no behavioral sexual selection was possible either among males or among females.
- A key to our experimental treatments was the elimination of coercion of females from simultaneous interactions with multiple males, that is, in all treatments except for lifelong virgins, females’ exposures to males were limited daily to only one male, so that no female ever saw more than one male in a 24‐hr period thereby eliminating opportunities for male–male behavioral competitive effects on subject females’ reproductive decisions.
- We randomly placed females in five treatments and males in one treatment at the start of the experiment. Each subject was the same age, and each treatment set began on the same day, so that the ages of all individuals in all treatments were the same.
- There were five treatments for females in the experiment and one for males, which we labeled arbitrarily as “A,” “B,” “C,” “D,” “E,” and “F” to mask the manipulation from helpers (Table 1).
- We then matched 150 females–30 per treatment–labeling each female subject with an ID # from 1 to 30, and 30 virgin males subjects. For a given treatment, we labeled vials with the treatment and a given ID # (e.g., A 1‐30, B1‐30, C1‐30, D‐1‐30, E 1‐30, E 1‐30, F 1‐30).
- We then sorted vials into 30 matching sets by ID numbers containing a single vial from each treatment. Thus, each matching set had six vials, one from each treatment but having the same ID #. Matching by ID number across treatments controlled for bench effects as we rotated the orientation of vials daily in boxes and on shelves. This matching of subjects between treatments also allowed us to do robust day by day comparisons of components of fitness among treatment females and of lifespans of females and males, similar to other published studies (Gowaty et al., 2010; Turner & Anderson, 1983)
- Our analyzes are of two types. Most tested a priori planned predictions of hypotheses (Leek & Peng, 2015), and thus, the results are capable of rejecting or confirming a priori predictions. In addition, as an explicit control, we characterize some of our analyzes as descriptive and exploratory. We define exploratory analyzes following (Leek & Peng, 2015/p 1314) as “data interpretation that builds on a descriptive analysis by searching for discoveries, trends, correlations, or relationships between the measurements to generate ideas or hypotheses.”

### 2.6 Treatments

See Table 1. On 9/8/2012, using flies 6‐day posteclosion, we randomly put 150 female subjects (30 individuals in each of five treatments) and 120 males (30 individuals used in four of the female treatments). We also put 30 additional males into a male only treatment (Table 1). We emphasize again that no posteclosion female or male saw more than one opposite sex conspecific on any day, eliminating any sexual selection from female–female and male–male behavioral competition, similar to an earlier study using *D. pseudoobscura* (Gowaty et al., 2010).

### 2.7 Behavioral observations

On day 1, we scanned all vials for 3 hr after placing a male in a vial with a female to record if the pairs copulated. Only nine of 150 female subjects each of whom were with a single male failed to copulate in the first three hrs of day 1: *M*_OC_ females: C14, C15, C26, C27, C30; and *M*_L_ females: D4, D17, E1, and E9. However, all females copulated on day 1, as offspring eclosed from each day‐1 vial. On following days, after moving females each day to new vials we scanned each vial for copulations. We emphasize that after day‐1, our attention was only on whether a subject had additional copulations. Our haphazard observations of copulations after the first day indicate that additional copulations occurred in all treatment groups in which males were present, however, we did not continue to watch vials throughout the 24 hr that females had access to males simply because it would have been impossible given the size of the experiment, not to mention extraordinarily costly. We emphasize that despite the interests of others in the numbers of copulations that polyandrous females might have, our interest was rather on the likelihood that females flexibly take or resist options for re‐mating (Gowaty, 2013). We designed the experiment to enhance females’ abilities to manage or avoid coercion from males that can arise under male–male behavioral sexual selection (Trivers, 1972): No female subject ever was with more than one male a day except for *M*_OC_ females who saw one male on the first day of the experiment and *V*_F_ females, who never encountered a male. Otherwise females had ad libitum access to interactions with a known male (*M*_L_) or a novel male each day (*P*_E_ or *P*_V_).

### 2.8 Components of fitness and numbers of observations

Each day before gently aspirating living subjects to new food vials, we recorded if subjects were alive or dead. We counted eggs in the previous day's vial and held it until eclosions occurred, noting the date and the number of eclosed adult offspring from each vial. We discarded all eclosed offspring, retaining the vial for a further 8 days, checking each day for additional eclosion. Three *V*_L,_ 2 *P*_V_, and 1 *P*_E_ females escaped. Survival analyzes described below excluded the six lost females; however, we retained observations of RS variables that were complete up to the day a female in a matched set was lost, because we used analyzes of treatment means over days to evaluate treatment differences. The mean number of oviposition days/female was 29.8 ± 14.0 (*SD*); maximum number of oviposition days/female was 56, the minimum 2. We recorded egg number from 4,474 unique vials. Egg number ranged from 0 to 100/d/female. Same‐day eggs eclosed over 2–6 days. Development time (oviposition to eclosion date) was 8–16 days. Recording of daily RS per female produced 6,697 unique observations.

*Statistical analyzes of* a priori *planned tests* (Leek & Peng, 2015) of hypotheses compared predictions of lifespan and components of reproductive success of female *V*_L_ versus *M*_OC_, *M*_OC_ versus *M*_L_, *M*_L_ versus *P*_E_, and *P*_E_ versus *P*_V_.

For tests of lifespan variation between treatments, we used the non‐parametric Kaplan‐Meier Log Ranks test emphasizing longer survival times, and the generalized Wilcoxon chi‐square test emphasizing early survival times.

To compare components of fitness between treatment pairs over female lifespans, we used differences in means/day/treatment to compare number of eggs, eclosed adult offspring, and arcsine‐transformed fraction of egg‐to‐adult survival (Table 1), similar to a study of *D. pseudoobscura* (Gowaty et al., 2010). Conclusions came from comparisons between two treatment means/day, with *df* = days − 1, which reduces expected bias from repeated measures. Means/day included fitness components for up to 60 females (30 per treatment). We included in our sign tests the average per day difference per treatment pairs over all days in which females from each treatment remained alive: *V*_L_ versus *M*_OC_, *M*_OC_ versus *M*_L_, *M*_L_ versus *P*_E_, and *P*_E_ versus *P*_V_. We tested if the average/day difference was significantly different from zero (Wilcoxon signed rank test with *df* = d − 1 and in which there were as many as 30 females in each treatment).

Even though the sign test statistical approach above reduces expected bias from repeated measures, we also evaluated the effect of repeated measures over days of female RS, using a mixed effect ANOVA to calculate the amount of variance contributed by repeated measures of individual females. The ANOVA modeled repeated measures over the days of female life and characterized effects on offspring viability of treatment, female age, and female age × treatment: All effects were significant (*p* < .0001), and the co‐variation within females over days was slight 0.031% ± .004 (*SD*) suggesting that our design was robust to any biases produced by measuring the RS of female subjects daily over their lives. The results of the mixed effects ANOVA failed to estimate treatment means for times after all *P*_V_ females were dead, thereby obscuring for other treatments the daily differences that were of most interest to us. We, therefore, report only the results from the matched treatments difference score sign test analyzes.

We note that in order to have completely randomized measures of female age and treatment, one would need to include only 1 day's observation of a female, while nevertheless retaining all females for life moving them through each treatment protocol. Maximum lifespan for females in this experiment was 62 days. Thus, to have completely independent samples from each day of life for, say, 30 females would require running an experiment with 1,830 subject females (plus males) from which one could randomly draw without replacement a set of unique females for each day of life. Such an experiment would be difficult requiring extraordinary resources, especially given the many controls we used.

*Unplanned analyzes allowed exploration* (Leek & Peng, 2015) of lifespan and RS variation as well as between‐treatment variances of fitness components.

We completed all statistical tests using JMP‐Pro 11 and we set the a priori significance level at ≤0.05.

## 3 RESULTS AND DISCUSSION

Figures 1, 2, 3, 4 show results for specific predictions between treatments. Table 2 summarizes predictions and results of a priori planned tests of hypotheses (Gowaty, 2012, 2013; Gowaty et al., 2010). Exploratory analyzes were of: (1) comparative lifespan variation among females (Figure 5a) and among males (Figure 5b); (2) mean changes over female lifespan in components of RS (Figure 6); and (3) variances in female RS and survival (Table 3).

### 3.1 *V*_L_ versus *M*_OC_

Lifespan variation of *V*_L_ versus *M*_OC_ females tested the hypothesis that copulation enhances female survival, a conclusion in a study of wild‐living *D. melanogaster* (Markow, 2011)*:* previously mated females lived longer than never‐mated females, a surprising result because mated individuals often die faster than virgins (Partridge, 1987). Markow speculatively attributed her unexpected finding either to (1) the enhanced feeding opportunities of already‐mated females, who were presumably older than unmated females and/or to (2) male‐derived benefits delivered at copulation. In the current experiment, subjects entered the experiment at the same age, yet *M*_OC_ females lived significantly longer than *V*_L_ females (Figure 1a) and produced significantly more eggs. Our experimentally controlled food availability plus the fact that all subjects were the same age put the differential feeding time idea off the table as an explanation of longer life in *M*_OC_ compared with *V*_L_ females. Some may argue that in our captive flies mated females were hungrier and ate more than *V*_L_ females enhancing the health of *M*_OC_ females, but others would expect that enhanced eating would decrease female lifespan (Grandison, Piper, & Partridge, 2009). Nevertheless, our results agree with Markow's, 2011) observations of wild flies. The significant enhancements to lifespan and egg number (Figure 1b) for *M*_OC_ compared with *V*_L_ females are consistent with ejaculate contributions nourishing zygotes and females (Gillott, 2003) and/or mating‐induced female resource contributions and/or immunity (Morrow & Innocenti, 2012; Zhong et al., 2013), but of course, our results cannot discern between these alternatives. The possibility of male‐derived benefits from copulation implies between‐sex physiological cooperation that may enhance mother's health, in contrast to male manipulation/coercion of females that may decrease female survival (Wigby & Chapman, 2005). The fact that a single copulation enhances female lifespan compared to lifespan of virgin females is consistent with the idea that male‐derived benefits may favor female multiple mating.

### 3.2 *M*_OC_ versus *M*_L_

*M*_OC_ and *M*_L_ (Figure 2) differences evaluated the cost of multiple copulations and tested female RS variation associated with possible sperm limitations (not enough or nonviable sperm), which occurs in some species (Turner & Anderson, 1983, 1984). Multiple copulations may be energy and time taxing for females, predicting that compared to *M*_OC_ females, *M*_L_ females die faster. Indeed, *M*_OC_ females lived significantly longer than *M*_L_ females (Figure 2a). Despite shorter lifespans, *M*_L_ females laid significantly more eggs, and produced significantly more eclosed offspring with significantly enhanced offspring viability (Figure 2b), all results consistent with the hypothesis that multiple copulations provide material benefits to females that enhance all components of female RS. Studies of *D. pseudoobscura* (Gowaty et al., 2010; Turner & Anderson, 1983, 1984) had similar results. Despite the survival costs to females of more than one copulation, the reproductive benefits to females of multiple copulations are suggestive of similar benefits from copulation with multiple mates, not just of multiple copulations, an idea which the next comparisons between *M*_L_ and *P*_E_ females directly tests.

### 3.3 *M*_L_ versus *P*_E_ females

Polyandry costs may not be offset by any benefits if encounters are random and mate choice is absent. Microbes are common, so that females mating with multiple partners are likely to have greater exposure to pathogenic fungi, viruses, and bacteria (Otti, 2015), which can affect female health and induce perhaps costly upregulation of immune responses in females (Knell & Webberley, 2004; Lockhart, Thrall, & Antonovics, 1996; Zhong et al., 2013) permitting the prediction that polyandrous females have great mortality risk than females with multiple copulations in lifelong monogamy (Figure 3). Frequently predicted benefits (Lively, 1996; Williams, 1975) of polyandry compared to lifelong monogamy include enhanced offspring viability from diversification of progeny genes (see summary, Table 2).

*M*_L_ and *P*_E_ females had statistically similar lifespans (Figure 3a), not a unique result (Gowaty et al., 2010; Simmons & Holley, 2011), but perhaps unexpected given that *P*_E_ females were with an unfamiliar, novel male each day, who had also been previously exposed to other females (except for the first day of the experiment) presumably increasing pathogen exposure risk. Offspring viability was significantly greater for *P*_E_ than *M*_L_ females (Figure 3b): Polyandry compared to monogamy enhances lineage success, reducing extinction risk (Price et al., 2010). There were no statistical differences in the number of eclosed offspring (2nd panel Figure 3b), but the distribution of daily differences showed *P*_E_ females had late life RS advantage over *M*_L_ females that seemed to have early life advantage over *P*_E_ females. Differences in offspring viability (3rd panel, Figure 3c) occurred because *M*_L_ females laid significantly more eggs, fewer of which survived than *P*_E_ females, a result not explained by negative density because the number of eggs and number of eclosed offspring were significantly positive in both treatments (*M*_L_ *r* ^2^ = 0.84, *p* < .0001 and for *P*_E_ *r* ^2^ = 0.96, *p* < .0001). The differences indicate an advantage in egg number for *M*_L_ females at younger ages and *P*_E_ females at older ages, therefore, given our interest in offspring viability, we truncated the comparison of offspring viability to subjects less than 43 days old: On 28 of 36 days difference scores were positive indicating greater egg‐to‐adult survival for *P*_E_ than *M*_L_ females. Assuming that *M*_L_ females had stronger constraints on mate choice than *P*_E_ females, the over‐lifetime observations of *P*_E_ advantage over *M*_L_ are consistent with the hypothesis (Anderson et al., 2007; Gowaty, 2008; Gowaty et al., 2007a) saying that females breeding under constraints compensate for expected deficits in health of offspring by increasing egg number.

The next comparison between polyandrous females with exposure to age‐matched experienced males *P*_E_ versus those exposed each day to virgin males *P*_V_, under the assumption that experienced males are less coercive than virgin males, directly tests the prediction that reduction in coercion benefits females.

### 3.4 *P*_V_ versus *P*_E_

Assuming inexperienced, young, virgin males are more sexually assertive (Hoffmann, 1990) than experienced, older, previously mated males, a comparison of polyandrous females daily with a new, young virgin male (*P*_V_) versus polyandrous females with an experienced, previously mated male age‐matched to the females (*P*_E_) may illustrate how behavioral variation of males to females (Long, Markow, & Yaeger, 1980) may affect female survival and RS. Further, given the classical assumption that females are unlikely to increase RS when they mate with multiple males, RS components should be no different for *P*_V_ versus *P*_E_ females.

*P*_V_ lifespan began declining when other females still had half or more of their lives in front of them. *P*_E_ females live significantly longer than *P*_V_ females (Figure 4a), and every RS component was significantly enhanced for *P*_E_ versus *P*_V_ females (Figure 4b, all three panels). Constant exposure to young, virgin males extracted costs to females and lowered offspring viability. As in *D. bipectinata* (Krishna, Santhosh, & Hegde, 2012), polyandrous *D. melanogaster* females mating older males had more offspring and healthier offspring than females mating younger males. Coercive attention from young males may explain female preferences for older (Avent, Price, & Wedell, 2008; Brooks & Kemp, 2001; Hansen & Price, 1995; Somashekar & Krishna, 2011), perhaps more sedate males, and not be just a function of male fertility that increases as males age (Long et al., 1980). We speculate that (1) differences will be revealed with comparisons of behavior of virgin versus already‐mated males to virgin and mated females (one on one to control for within‐sex behavioral competition) and (2) mechanistic studies will reveal physiological loads with impacts on female's RS when they interact exclusively with eager, and perhaps lower “fertility” virgin males.

Table 2 summarizes the a priori predictions and the results in Figures 1, 2, 3, and 4.

### 3.5 Exploratory analyzes

The experiment facilitated several exploratory analyzes, including across treatment comparison of the lifespans among females (Figure 5a) and among males (Figure 5b). Behavioral sexual selection could not explain the variation among females (Figure 5a) or among males (Figure 5b), because no subject was ever with a same sex conspecific in this experiment.

That *P*_V_ females (Figure 5a) died significantly faster than females in other treatments suggests usually over‐looked benefits to females who–in the wild–may be able to escape virgin males, just by flying away.

Despite absence of male–male behavioral competition, multiply–mated males (those that rotated between *P*_E_ females) died significantly faster than males in other treatments (Figure 5b). We discuss the comparative lifespan variation between subject females and males elsewhere (Gowaty et al. in prep.).

The mean daily variation in RS components with the time course of females’ lives (Figure 6a–c) shows a downward slope over all treatments in egg number (Figure 6a) suggesting intrinsic female resources available for egg production decline with female age in *D. melangaster* independent of their mating history, even when resource availability is ad libitum with food amounts identical and controlled over female lifespans, days of the experiment, and treatments. Similarly, numbers of eclosed offspring (Figure 6b) decline in all treatments with female age. In contrast, offspring viability (Figure 6c) shows treatment variation that declined precipitously for *M*_OC_ females when they are about 20 days old, but did not occur for *M*_L_ females until they are about 40 days old, a benefit most likely due to multiple copulations and perhaps benefits from seminal contributions from the pair male. In contrast, offspring viability of *P*_E_ females remained higher throughout their lives, a benefit from mating with multiple males, even though the *P*_E_ males aged as the *M*_L_ males did. At about 20 days, offspring viability for *P*_V_ females started to decline perhaps due to behavioral “eagarness” females experienced from virgin males. The rapid decline in offspring viability for *M*_L_ females compared to *P*_E_ females also suggests a potential interaction effect with males’ reproductive capacities, when with one female for life versus with several females over their lifespans, suggesting significant mating costs to both sexes in monogamy.

Significant variance differences between treatments (Table 3) included female survival (*p* < .001), egg number (*p* < .0153), and offspring viability (*p* < .0001). Variance differences between females in lifespan, egg number, and offspring viability are consistent with some social environments having greater potential for producing evolutionary responses in females, an observation begging for more investigation of selection on females.

## 4 CONCLUSIONS

The results of these experiments provided no support for nonadaptive, by‐product polyandry (Table 2). All other functional a priori hypotheses had some support: Compared to virginity, one copulation may enhance female survival–at least in the absence of the possibility of behavioral competition of males, a prospect for further study from proximate and ultimate perspectives. However, a single ejaculate was seldom enough for fertilization of a lifelong supply of eggs, providing an adaptive explanation for female multiple copulations via polyandry or lifelong monogamy (which may be very hard for female flies to achieve).

*M*_L_ females oviposited significantly more eggs than *P*_E_ females, but there were no statistical differences in the numbers of eclosed offspring. Thus *P*_E_ females had statistically greater offspring viability indicating that polyandry fosters lineage success (Price et al., 2010). *P*_E_ females with lifelong random exposure to many males–one at a time–had opportunities to manage sperm use, which also begs greater mechanistic attention.

The comparisons between *P*_V_ and *P*_E_ females seem completely consistent with the idea that virgin males are more “eager” compared with more “sedate” experienced males: *P*_V_ females have significantly lower fitness than *P*_E_ females, a result consistent with previous observations of eager virgins versus more sedate experienced males (Greenspan & Ferveur, 2000). An experiment that would potentially buttress that conclusion would include detailed behavioral observations of subject females and the males they are exposed to every day of their lives. Even without such labor‐intensive observations our results also are consistent with the earlier observations of (Hoffman 1990) indicating the enhanced “eagerness” of virgin males compared with experienced males. Our results are consistent too with the idea that females who are able to escape male harassment may have longer lives and greater reproductive success at all ages than females unable to escape male coercion.

The evidence here suggests that greater female control via reduced exposures to simultaneous male–male behavioral competition is adaptive for *D. melanogaster* females. Wild females who can fly away may seek coercion‐free social situations, flexibly favoring their own continued survival and the viabilities of their offspring (Gowaty, 2013). Polyandry may have fewer benefits for laboratory‐living than wild‐living flies simply because opportunities for avoidance of coercion are fewer inside of fly‐filled jars than outside.

### 4.1 Differences with an earlier study of D. melanogaster polyandry

In contrast to the results here**,** Brown, Bjork, Schneider, and Pitnick (2004) found no benefits for polyandrous females compared to monogamous females with multiple copulations. The differences in scope and methods of the two studies probably completely account for the differences in results. Brown et al.'s source populations had been in laboratory culture for 200 +  generations allowing for significant selection in captivity, ours for <20. Ours were free of the endosymbionts, *Spiroplasma* and *Wolbachia*, something not reported in Brown et al. Their experiment lasted 14 days when their subjects were 19–20 days old; ours lasted 69 days including the natural lifespan of each of our subjects who each entered our experiment when they were 6 days old. Their monogamous subjects were exposed to the same male at 48‐hr interval for 4 hr each time, and their polyandrous females saw a new unfamiliar male every other day (without notes about ages or experience of the males). In contrast, our matched pairs–*M*_L_ and *P*_E_–were constantly exposed to males (but only one at a time). Our repeated measures allowed comparisons of daily variation in RS of all subjects, and controlled uninteresting variance due to bench effects. Last, our study used only wild‐type flies, while Brown et al. used flies with visible heritable mutations to evaluate sperm competition, a part of their study with no counterpart in ours. Any of the many differences in source of flies, endosymbiont‐load variations, culturing techniques, handling protocols, time in captivity, use of mutants versus wild‐type flies, methods including timing of mating, handling variation, etc. could account for the differing conclusions between Brown et al. (2004) and the current study.

## CONFLICT OF INTEREST

None declared.

## AUTHOR CONTRIBUTIONS

PAG designed experimental treatments. SC captured, cultured, and maintained populations, carried out all treatments, managing helpers, daily data acquisition, and computerization of data. SC and PAG checked computer records against manuscript records. BCF designed and ran genetic tests for endosymbionts. PAG and WCB analyzed the data and PAG wrote the manuscript. PAG was corresponding author.
